# Factors Associated with 30- and 90-Day Mortality After Percutaneous Cholecystostomy for Acute Cholecystitis: A Single-Centre Retrospective Cohort Study

**DOI:** 10.3390/jcm15166270

**Published:** 2026-08-13

**Authors:** Berkan Acar, Ali Muhtaroğlu, Elif Yılmaz, Serdar Acar, Yavuz Çekiç, Mehmet Yıldız

**Affiliations:** 1 Department of General Surgery, Faculty of Medicine, Giresun University, 28200 Giresun, Türkiye; berkanacar01@gmail.com; 2 General Surgery Clinic, Private Adatıp Hospital, 54050 Sakarya, Türkiye; 3 General Surgery Clinic, Private Umut Hospital, 52200 Ordu, Türkiye; ylmzelif24@gmail.com; 4 Department of General Surgery, Faculty of Medicine, Süleyman Demirel University, 32260 Isparta, Türkiye; dr.serdaracar@gmail.com; 5 School of Surgery, Yorkshire and Humber Deanery, NHS England, Leeds LS2 9JT, UK; yavuz.cekic@nhs.net; 6 Giresun Provincial Health Directorate, Bulancak Sehit Er Enver Erdogan Family Health Center, 28300 Giresun, Türkiye; drmehmetyildiz54@gmail.com

**Keywords:** acute cholecystitis, percutaneous cholecystostomy, early mortality, mortality-associated factors, Tokyo severity grade, intensive care, high-risk surgical patients

## Abstract

**Background/Objectives:** Percutaneous cholecystostomy (PC) is used for acute cholecystitis when early cholecystectomy is considered unsafe. We evaluated factors associated with 30- and 90-day mortality after PC. **Methods:** This single-centre retrospective cohort included 84 consecutive patients treated between December 2021 and December 2025. Clinical characteristics, comorbidity, Tokyo severity grade, intensive care unit (ICU) admission during the index episode, microbiology and admission laboratory values were analysed. Separate age-adjusted logistic models included age and one pre-specified factor at a time. **Results:** The median age was 74.5 years, and 50 patients (59.5%) were male. Thirty-one patients (36.9%) had Tokyo grade III disease and 26 (31.0%) were admitted to the ICU during the index episode. The thirty-day mortality was 12/84 (14.3%; the 95% confidence interval [CI], 8.4–23.3). The crude 90-day mortality was 20/84 (23.8%; 95% CI, 16.0–33.9), and mortality among 82 patients with complete 90-day status was 20/82 (24.4%; 95% CI, 16.4–34.7). In separate age-adjusted models, ICU admission during the index episode was associated with 30-day (odds ratio [OR], 14.31; 95% CI, 2.77–73.99) and 90-day mortality (OR, 21.10; 95% CI, 5.65–78.85); Tokyo grade III disease showed corresponding associations (OR, 9.44; 95% CI, 1.84–48.35 and OR, 19.07; 95% CI, 4.74–76.66). **Conclusions:** Tokyo grade III disease and ICU admission during the index episode were strongly associated with early mortality. These estimates do not demonstrate independent effects or validated predictive performance and require confirmation in larger multicentre cohorts.

## 1. Introduction

Acute cholecystitis is generally treated with early laparoscopic cholecystectomy when the patient can tolerate surgery. Percutaneous cholecystostomy (PC) is reserved mainly for patients in whom anaesthesia and early surgery are considered unacceptably hazardous because of severe infection, organ dysfunction, cardiopulmonary instability, comorbidity or limited physiological reserve.

The course after PC is heterogeneous. Some patients recover and later undergo interval cholecystectomy, whereas others remain unsuitable for definitive surgery or die early despite undergoing technically successful drainage. A recent systematic review therefore emphasised both appropriate patient selection and structured post-drainage management [1]. Huang et al. likewise reported substantial 30-day mortality, particularly in patients treated in intensive care, and identified bilirubin and estimated surgical risk as useful markers [2].

Risk assessment in this setting commonly incorporates the American Society of Anesthesiologists physical status classification, Charlson comorbidity index, Acute Physiology and Chronic Health Evaluation II (APACHE-II) score and Tokyo severity grade. Tokyo Guidelines 2018 grade has shown value for outcome stratification [3], while comorbidity burden, albumin and surgical-risk measures have also been linked to survival and subsequent interval cholecystectomy [4,5]. These measures capture related domains and should not be interpreted as interchangeable or causal.

Age alone is an incomplete measure of vulnerability. Studies involving older patients indicate that timing of intervention, nutritional and inflammatory status, comorbidity and severity of presentation also influence outcome [6]. Readily available laboratory measures, including white cell count, C-reactive protein, renal indices, bilirubin and inflammatory ratios, may provide additional context, although exploratory comparisons are vulnerable to multiplicity and confounding [7].

Interpretation must also account for treatment selection: patients who later undergo cholecystectomy have already survived the acute episode and recovered sufficiently to be selected for surgery. We therefore conducted a single-centre retrospective cohort study to examine clinical and laboratory factors associated with 30- and 90-day mortality after PC. The aim was to describe clinically useful associations, not to develop or validate a prediction model.

## 2. Materials and Methods

### 2.1. Study Design and Setting

This was a single-centre retrospective cohort study of consecutive adult patients who underwent PC for acute cholecystitis after being considered unsuitable for early cholecystectomy. The study was conducted at Giresun University Training and Research Hospital, a tertiary centre with emergency general surgery, hepatopancreatobiliary surgery, interventional radiology and intensive care services. Patients were identified by the date of cholecystostomy insertion, and the study period extended from 16 December 2021 to 24 December 2025.

The retrospective protocol was approved before analysis by the Giresun University Medical Research Ethics Committee (decision no. 01.04.2026/07, dated 1 April 2026; written notification dated 8 April 2026). The decision letter bears the protocol title concerning systemic inflammatory markers and bile-culture positivity in patients undergoing PC; the present mortality study is a secondary analysis of the same approved retrospective cohort. Owing to the retrospective design and use of anonymised clinical data, the requirement for individual informed consent was waived. The study was prepared in accordance with the Strengthening the Reporting of Observational Studies in Epidemiology (STROBE) statement and was not prospectively registered.

### 2.2. Patient Selection

All adult patients who underwent PC for acute cholecystitis during the study period were screened. Patients were included if they had a diagnosis of acute cholecystitis supported by clinical, laboratory and radiological findings, underwent percutaneous gallbladder drainage and were deemed unsuitable for early cholecystectomy by the treating team during the index episode.

Early cholecystectomy was considered unsuitable when the anticipated operative or anaesthetic risk was judged unacceptable because of advanced age, comorbidity, organ dysfunction, sepsis, cardiopulmonary instability, intensive care needs, poor physiological reserve, anticoagulation-related concern or another documented clinical factor. Decisions were made by the attending surgical team with anaesthesia, intensive care and interventional radiology input when required. No mandatory institutional score or single threshold was used; American Society of Anesthesiologists (ASA) physical status, formal frailty assessment and APACHE-II/Sequential Organ Failure Assessment (SOFA) scores were not consistently documented and therefore could not be applied uniformly.

Patients were excluded if cholecystostomy had been performed for an indication other than acute cholecystitis, if the diagnosis of acute cholecystitis could not be confirmed from the medical record or if essential mortality data were unavailable. The final study cohort comprised 84 patients.

### 2.3. Diagnosis and Severity Assessment

The diagnosis of acute cholecystitis was based on the overall clinical picture, including right upper quadrant findings, systemic inflammation and imaging evidence of gallbladder inflammation. Imaging was performed using ultrasonography, computed tomography or magnetic resonance cholangiopancreatography according to clinical need and local practice.

For the descriptive analysis, acute cholecystitis presentation was assigned to one of three mutually exclusive categories. Any case with documented gallbladder perforation was assigned to the perforated category irrespective of stone status; non-perforated cases were classified as calculous or acalculous. This hierarchy avoided double counting while recognising that perforation can coexist biologically with either aetiology. Disease severity was graded as Tokyo grade I, II or III; grade III was compared with grades I-II in the principal analyses.

ICU admission was defined as any admission to intensive care during the index acute-care episode. Whether ICU admission occurred during that episode was linked to Tokyo severity grade at the patient level. However, its timing relative to PC could not be categorised reproducibly as before, contemporaneous with or after drainage. ICU admission was therefore treated as an index-episode severity marker rather than a uniformly pre-procedural or baseline factor.

### 2.4. Percutaneous Cholecystostomy and Clinical Management

PC was performed for gallbladder decompression and source control using standard percutaneous image-guided techniques according to institutional practice. Technical success was defined as completed catheter placement within the gallbladder with aspiration or drainage of gallbladder contents. All 84 included records documented a completed PC procedure; because cohort inclusion was based on completed drainage, this figure should not be interpreted as a success rate among all attempted procedures. Access route, guidance modality, catheter calibre, time from admission to drainage and procedure-related complication grading were not retained with sufficient completeness in the final analytical dataset for uniform reporting.

All patients received supportive care, analgesia, fluid resuscitation and antimicrobial therapy as clinically indicated. Empirical antibiotics were selected according to local practice and modified when clinical response, microbiological findings or antimicrobial susceptibility results supported a change in treatment. Bile or gallbladder aspirate was sent for culture when obtained during drainage. Culture results were recorded as positive, negative or unavailable.

### 2.5. Data Collection

Data were extracted retrospectively from electronic medical records, laboratory systems, imaging reports, microbiology reports, interventional radiology documentation, operative records, discharge summaries and follow-up notes.

The final patient-level analytical dataset contained linked values for age, sex, Charlson comorbidity index, Tokyo severity grade, symptom duration, ICU admission during the index episode, the mutually exclusive cholecystitis presentation category, hospital stay, catheter duration, bile culture result, antimicrobial treatment, interval cholecystectomy and survival status. Individual comorbidity categories (including diabetes mellitus), chills, previous biliary interventions, concurrent acute pancreatitis, ASA physical status, formal frailty measures, APACHE-II/SOFA scores and serial procalcitonin were not sufficiently complete or standardised for valid cohort-level analysis and were not imputed.

Admission laboratory values were recorded as candidate exploratory variables. These included white cell count, haemoglobin, haematocrit, neutrophil percentage, lymphocyte percentage, platelet count, urea, creatinine, sodium, potassium, calcium, alanine aminotransferase, aspartate aminotransferase, alkaline phosphatase, gamma-glutamyl transferase, total bilirubin, direct bilirubin, indirect bilirubin, amylase, lipase, lactate dehydrogenase and C-reactive protein. The neutrophil-to-lymphocyte ratio was calculated from the differential white cell count. The platelet-to-lymphocyte ratio was not used as a primary variable unless it could be recalculated using the absolute lymphocyte count.

### 2.6. Outcomes and Candidate Variables

The primary outcomes were 30-day and 90-day all-cause mortality, calculated from the date of PC insertion. Overall survival was the interval from PC insertion to death or last known follow-up. For the 90-day endpoint, administrative ascertainment extended through 24 March 2026, 90 days after the final PC in the inclusion period. Patients alive at last contact were censored on that date; those with personal follow-up shorter than the relevant endpoint were censored in time-to-event analyses and excluded from the primary binary comparison.

Candidate variables were selected prior to analysis on the basis of clinical relevance and published evidence. The principal variables were age, age ≥ 80 years, Charlson comorbidity index, Tokyo grade III disease, ICU admission during the index episode, symptom duration, cholecystitis aetiology, perforation, culture positivity and admission laboratory markers (white blood cell count, neutrophil count, lymphocyte count, neutrophil-to-lymphocyte ratio, C-reactive protein, urea, creatinine and total bilirubin).

Tokyo grade III disease and ICU admission during the index episode were the pre-specified clinical factors of principal interest because both reflect systemic severity and organ dysfunction. Their patient-level joint distribution was examined because these factors represent overlapping severity domains. Age was included as an adjustment variable. Laboratory comparisons were exploratory. Interval cholecystectomy was not treated as a baseline factor because patients who underwent surgery had already survived the acute episode and recovered sufficiently for operative selection.

### 2.7. Statistical Analysis

Statistical analysis was performed using Python version 3.13.5. Data handling and summary statistics were undertaken with pandas version 2.2.3 and NumPy version 2.3.5. Statistical testing was performed with SciPy version 1.17.0 and statsmodels version 0.14.6. Figures were generated using Matplotlib version 3.10.8. The analysis code is available from the corresponding author on reasonable request.

As this retrospective cohort included all eligible consecutive patients treated during the study period, no formal sample size calculation was performed. Continuous variables were assessed for distribution by visual inspection of histograms and quantile–quantile plots. Because most clinical and laboratory variables were not normally distributed, continuous data are presented as medians with interquartile ranges. Categorical variables are presented as numbers and percentages.

For univariable comparisons, continuous variables were compared using the Mann–Whitney U test. Categorical variables were compared using Pearson’s chi-squared test when expected cell counts were adequate and Fisher’s exact test otherwise. Univariable ORs with 95% CIs were calculated for clinically important binary factors. Wilson 95% CIs were calculated for the 30- and 90-day mortality proportions.

Given the modest sample size and 12 deaths that occurred by 30 days, multivariable modelling was deliberately restricted. Stepwise selection was not used. Separate logistic models included age and one pre-specified factor at a time; the results are therefore described as age-adjusted associations, not independent effects. Patient-level cross-tabulation showed that all ICU admissions occurred among Tokyo grade III patients, leaving only five grade III patients who were not admitted to ICU. This nested distribution and the limited event count did not support a stable joint model, so Tokyo grade III disease and ICU admission were modelled separately. Continuous variables were entered on the scales shown in the tables. Formal linearity-in-the-logit diagnostics and Firth penalised-logistic sensitivity models were not performed; this is acknowledged as a limitation, and the estimates are interpreted as exploratory.

Overall survival was described using the Kaplan–Meier method. Survival curves were compared using the log-rank test when clinically appropriate. These analyses supported interpretation of early mortality patterns rather than construction of a formal prognostic score.

Analyses used available cases and no values were imputed. Thirty-day status was available for all 84 patients; 90-day status was available for 82. Among the 82 patients in the primary 90-day comparison, the clinical and laboratory variables displayed in Table 1 and Table 2 were complete, except bile-culture status, which was available for 69 patients. An extreme-case sensitivity analysis classified both patients with incomplete 90-day status first as alive and then as dead and recalculated the mortality proportion and crude associations for Tokyo grade III and ICU admission. All tests were two-sided. Laboratory and subgroup comparisons were exploratory; no multiplicity correction was applied, and isolated nominal *p* values were interpreted as hypothesis-generating.

## 3. Results

The study included 84 consecutive patients. The median age was 74.5 years (interquartile range [IQR], 65.0–87.0); 50 (59.5%) were male, 31 (36.9%) had Tokyo grade III disease, and 26 (31.0%) were admitted to ICU during the index episode. The thirty-day mortality was 12/84 (14.3%; 95% CI, 8.4–23.3). The crude 90-day mortality was 20/84 (23.8%; 95% CI, 16.0–33.9). Two patients were alive at last contact but had less than 90 days of documented follow-up; among 82 evaluable patients, the 90-day mortality was 20/82 (24.4%; 95% CI, 16.4–34.7). In an extreme-case analysis, the 90-day mortality proportion ranged from 20/84 (23.8%; both censored patients assumed alive) to 22/84 (26.2%; 95% CI, 18.0–36.5; both assumed dead). Baseline characteristics are shown in Table 1.

Patients who died within 90 days were older and had a higher comorbidity burden than those who were alive at 90 days (Table 1). Tokyo grade III disease and ICU admission during the index episode showed the clearest separation. The mutually exclusive cholecystitis presentation category and culture positivity were not associated with 90-day mortality.

Patient-level cross-tabulation confirmed that there was substantial overlap between Tokyo grade III disease and ICU admission. All 26 ICU admissions occurred among the 31 patients with Tokyo grade III disease; the remaining five grade III patients and all 53 patients with grade I–II disease were managed without ICU (Supplementary Table S1). Thus, ICU admission was fully nested within Tokyo grade III in this cohort. Because only five grade III patients were not admitted to ICU and the timing of ICU admission relative to drainage could not be reconstructed consistently, the incremental information provided by ICU admission beyond Tokyo grade could not be estimated reliably.

All 84 included patients had a completed PC procedure recorded. This reflects the inclusion definition and is not a technical-success denominator for all attempted procedures. Access route, guidance modality, catheter calibre, time to drainage and procedure-related complications were not sufficiently complete in the analytical dataset for valid frequency reporting.

Diabetes mellitus, chills at presentation, previous biliary interventions and concurrent acute pancreatitis were not sufficiently complete or standardised in the final analytical dataset for valid prevalence or mortality analyses. These variables were therefore not imputed, and no cohort-specific associations are claimed.

Admission laboratory values are summarised in Table 2. Non-survivors had higher urea, creatinine and total bilirubin. NLR was numerically higher among 90-day non-survivors, whereas white cell count did not differ materially. C-reactive protein (CRP) was lower among patients who died within 90 days; because this was one of numerous uncorrected exploratory comparisons, the isolated nominal association may be attributable to chance and is considered hypothesis-generating.

The stratified mortality pattern is presented in Table 3 and Supplementary Figure S1. Mortality rose stepwise with the Tokyo severity grade. Among patients with Tokyo grade III disease, the 30-day mortality was 10/31 (32.3%) and the evaluable 90-day mortality was 17/30 (56.7%); the corresponding rates among those with grade I-II disease were 2/53 (3.8%) and 3/52 (5.8%). For ICU admission during the index episode, the 30-day mortality was 10/26 (38.5%) versus 2/58 (3.4%), and the evaluable 90-day mortality was 16/25 (64.0%) versus 4/57 (7.0%). Age of at least 80 years, a Charlson comorbidity index of at least 2 and higher admission urea and total bilirubin were also associated with higher early mortality in descriptive stratification.

**Figure 1:**
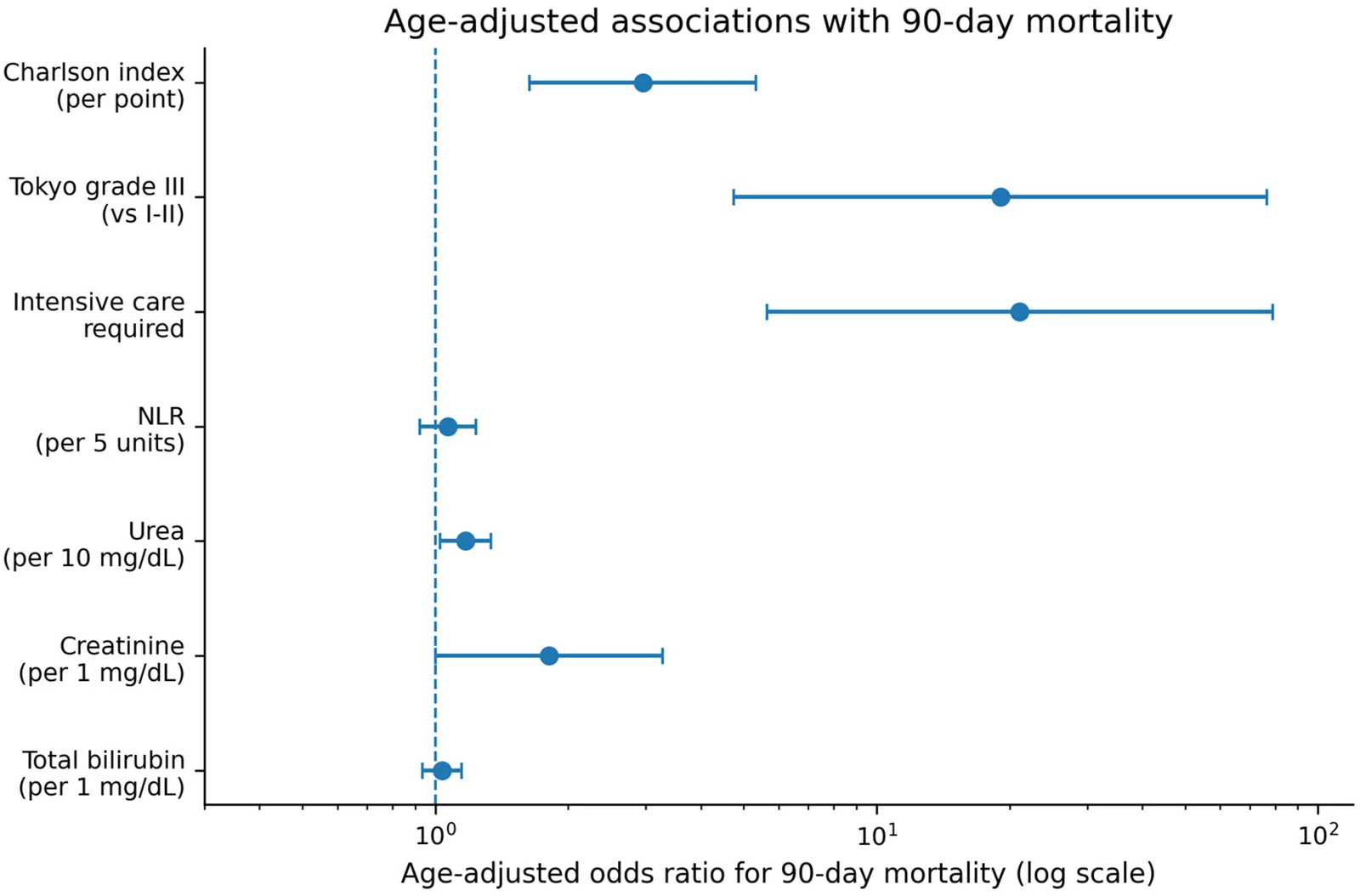
Age-adjusted odds ratios for 90-day mortality in separate age-adjusted models. Abbreviation: NLR, neutrophil-to-lymphocyte ratio.

Univariable and age-adjusted logistic associations are shown in Table 4 and Table 5, with age-adjusted 90-day associations displayed in Figure 1. Tokyo grade III disease and ICU admission during the index episode had the largest observed associations with early death. Because ICU admission was nested within Tokyo grade III, these were separate models containing age and one factor at a time and should be interpreted as alternative severity associations, not mutually adjusted or independently validated effects. Higher Charlson index and admission urea also showed age-adjusted associations with 90-day mortality. Wide CIs reflect the limited event count. Extreme-case sensitivity analyses for incomplete 90-day status are shown in Table 6 and did not materially alter the crude associations.

**Figure 2:**
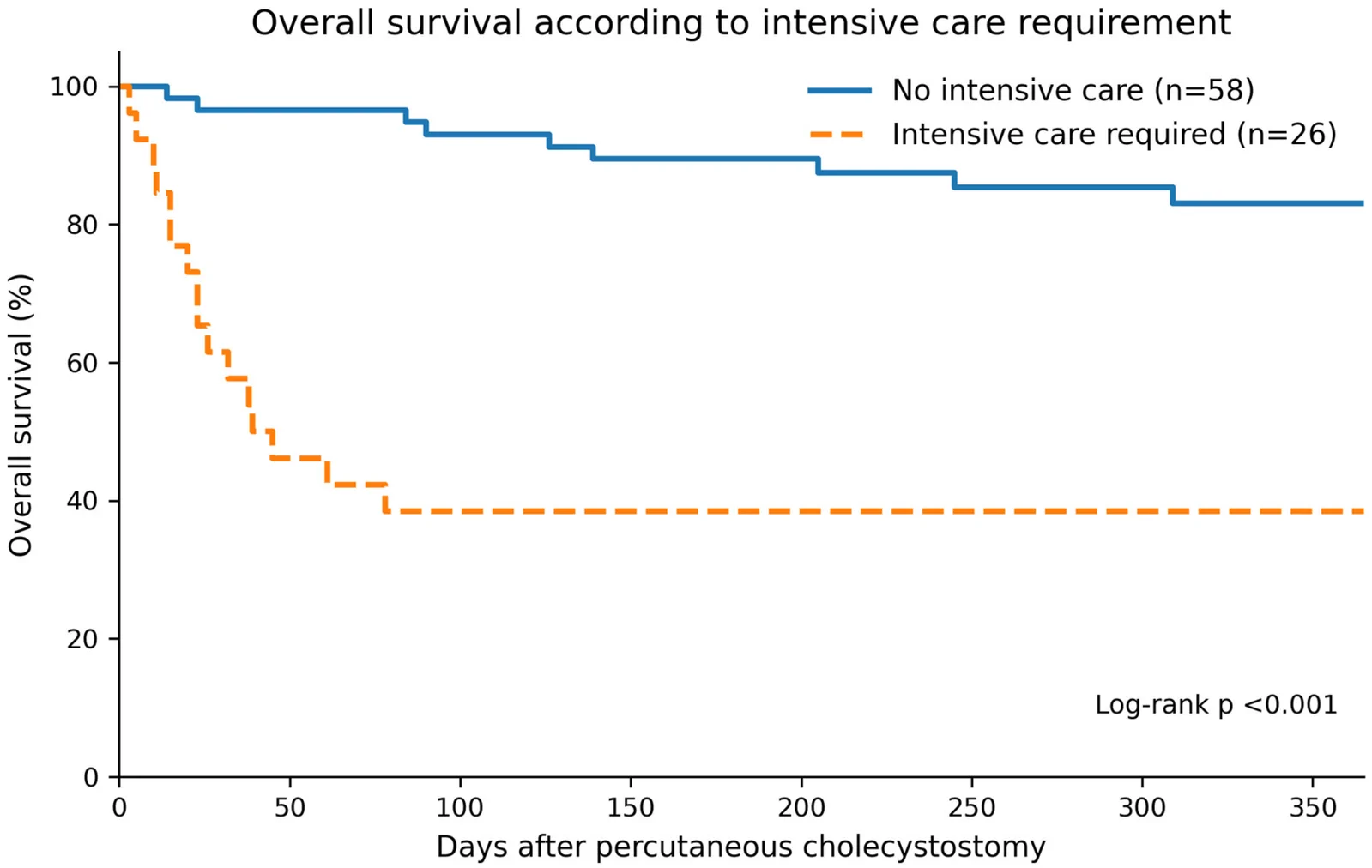
Kaplan–Meier survival according to intensive care unit admission during the index episode.

Kaplan–Meier curves showed early separation when patients were grouped by ICU admission at any time during the index episode (Figure 2). Estimated survival in the ICU group was 61.5% at 30 days and 38.5% at 90 days, compared with 96.6% and 93.0% in patients managed without ICU (log-rank *p* < 0.001). Because ICU admission was not uniformly pre-procedural, these curves are descriptive and do not provide evidence of baseline risk separation. A similar descriptive pattern was observed when survival was stratified by Tokyo grade III disease.

## 4. Discussion

In this single-centre cohort, Tokyo grade III disease and ICU admission during the index episode showed the largest associations with 30- and 90-day mortality. The reported models were adjusted for age only, one factor at a time; they do not establish independent prediction or causality. Older age, greater comorbidity, renal dysfunction and higher bilirubin provided additional context, while the wide CIs underscore the imprecision of this small cohort.

This distinction is important. The current literature generally supports cholecystectomy when patients can tolerate surgery, even in many older or high-risk groups. Recent comparative and meta-analytic work has shown better outcomes with cholecystectomy than with percutaneous drainage in broad patient populations with acute cholecystitis, although these comparisons are complicated by selection bias [8]. Large-scale data in older adults with multimorbidity also suggest that, when genuine clinical equipoise exists, operative treatment may reduce readmissions and emergency department revisits and may be associated with lower short-term mortality in risk-adjusted analyses [9]. These findings should not be read as an argument against PC. Rather, they emphasise the need to reserve it for the group in whom the risk of early surgery is truly prohibitive. A structured pathway helps protect operable patients from unnecessary drainage while preserving PC for those who need urgent source control but cannot safely undergo anaesthesia and cholecystectomy [10].

Thirty-day mortality was 14.3% (95% CI, 8.4–23.3), and 90-day mortality among evaluable patients was 24.4% (95% CI, 16.4–34.7). These rates are consistent with a cohort in which more than one-third had Tokyo grade III disease and almost one-third entered ICU during the index episode. Contemporary series vary substantially because of differences in age, comorbidity, disease severity, drainage indications and institutional thresholds [11,12].

Ninety-day mortality was 64.0% among patients admitted to ICU during the index episode and 7.0% among those managed without ICU. This is a clinically recognisable severity signal, but its timing matters: ICU admission could precede or follow drainage and could not be reconstructed consistently from the final analytical dataset. All 26 ICU admissions occurred among Tokyo grade III patients, and only five grade III patients were managed without ICU. The two factors therefore captured heavily overlapping, nested severity information in this cohort. Accordingly, the separate age-adjusted models should be interpreted as parallel descriptions of severe illness, not as evidence that ICU admission adds independent prognostic information beyond Tokyo grade.

Tokyo grade III disease showed a similarly powerful association with early death. Patients with grade III disease had 30-day mortality of 32.3% and 90-day mortality of 56.7%, compared with 3.8% and 5.8% among patients with grade I–II disease. This supports the clinical usefulness of severity grading in this setting. Tokyo grade III is not merely a label for a difficult gallbladder; it identifies acute cholecystitis complicated by organ dysfunction. PC can decompress the gallbladder and contribute to source control, but it cannot reverse all the consequences of systemic illness. The patients who died early were those in whom the acute biliary event occurred against a background of severe physiological stress.

Age also mattered, but it was not the whole story. Patients aged 80 years or older had higher 30- and 90-day mortality than younger patients, and non-survivors were older overall. Yet age alone is a blunt instrument. Some older patients recover well after drainage, whereas younger patients with organ dysfunction may do poorly. A more useful approach is to interpret age alongside functional reserve, comorbidity, disease severity and acute organ dysfunction. Older adult studies have similarly shown that outcomes after PC are closely related to baseline vulnerability rather than chronological age alone [12]. The same principle applies to the Charlson comorbidity index. A higher Charlson score was associated with worse 90-day survival in our analysis, but it should be understood as one component of risk rather than a stand-alone decision tool.

Diabetes mellitus was not available as a sufficiently complete, separately standardised variable in the final analytical dataset; a cohort-specific prevalence or mortality association could therefore not be estimated. Comorbidity burden was instead represented by the Charlson comorbidity index. This absence limits disease-specific interpretation.

Higher urea, creatinine and total bilirubin were associated with early mortality and may reflect renal stress, hypoperfusion, biliary obstruction, hepatic dysfunction or multi-organ illness. CRP was unexpectedly lower among 90-day non-survivors. This isolated result arose from numerous exploratory laboratory comparisons without multiplicity correction and may be a chance finding. It should not be considered evidence of a protective inflammatory response or used clinically without replication.

NLR was higher among 30-day non-survivors and numerically higher among 90-day non-survivors, but the age-adjusted estimates were imprecise and did not consistently exclude the null. This exploratory finding may reflect systemic inflammatory stress but does not establish information beyond Tokyo grade, ICU admission or renal function.

Concurrent acute pancreatitis and chills were not sufficiently consistently documented in the final analytical dataset to support valid prevalence or mortality analyses. Serial procalcitonin was likewise unavailable. Their absence from the reported analysis should not be interpreted as evidence that these clinical features were absent.

Microbiological culture positivity was not associated with early mortality. This does not mean that bile cultures are unimportant. They remain valuable for antimicrobial stewardship, particularly in patients with severe disease, intensive care admission, prior antibiotic exposure or poor clinical response. It does suggest, however, that culture positivity is not the same as systemic severity. A positive bile culture may guide treatment, but it does not necessarily identify the patient most likely to die. Future studies should go beyond culture positivity and evaluate antimicrobial concordance, multidrug resistance, extended-spectrum beta-lactamase production, vancomycin-resistant enterococci and timing of antibiotic modification.

The modelling strategy was deliberately conservative. With 12 deaths by 30 days and 20 deaths among 82 evaluable patients by 90 days, a larger model would have been unstable. The complete nesting of ICU admission within Tokyo grade III and the very small grade III/no-ICU subgroup made a joint model poorly supported. Separate age-adjusted models therefore estimated the association of one factor at a time. The large ORs for Tokyo grade III disease and ICU admission were accompanied by wide CIs and should not be presented as independent effects or a validated model.

The relationship between PC and later cholecystectomy also requires careful interpretation. In our cohort, only selected patients proceeded to interval cholecystectomy, and these patients were younger and less likely to have required intensive care. Their favourable survival should not be interpreted as proof that surgery caused better survival. Patients who undergo interval cholecystectomy have already survived the acute episode, recovered enough to be reassessed and been selected as fit for operation. Recent national data in grade III acute cholecystitis suggest that PC may serve as a useful bridge to later surgery in selected patients [13]. At the same time, large database work shows that many patients never reach interval cholecystectomy, particularly those with comorbidities such as chronic ischaemic heart disease, congestive heart failure, chronic obstructive pulmonary disease, ascites, diabetes or septic shock [14]. Interval surgery is therefore best viewed as a marker of recovery and selection, not a baseline factor.

The clinical implication is that PC should be the start of a structured decision process. Initial source control should be followed by risk assessment and evaluation for definitive surgery, catheter removal, further biliary intervention or non-operative or palliative care. Timing may also matter. Recent work has examined how cholecystostomy timing across acute cholecystitis grades relates to clinical outcomes [15]. Our dataset did not consistently capture time from diagnosis or admission to drainage, which is a limitation. Future outcome studies should include this variable because delayed source control may interact with disease severity and organ dysfunction.

A pathway-based approach is essential. Recent expert consensus work has emphasised that post-cholecystostomy management should be multidisciplinary and should include reassessment for bridge-to-surgery versus definitive drainage strategies [16]. Our findings support that view. Patients with Tokyo grade III disease or ICU admission during the index episode should not simply receive a catheter and be observed passively. They need early risk discussion, active optimisation, close review of clinical response and a clear plan for reassessment once sepsis and organ dysfunction improve. For some patients, this reassessment will lead to interval cholecystectomy. For others, it will appropriately confirm that definitive non-operative management is the safest course.

## 5. Strengths and Limitations

Strengths include the consecutive clinically high-risk cohort, clinically meaningful 30- and 90-day outcomes, transparent separation of interval surgery from baseline factors and a restrained modelling strategy intended to limit overfitting.

Limitations include the retrospective single-centre design; non-standardised selection for PC; residual confounding; and incomplete availability of individual comorbidities (including diabetes), ASA class, frailty, APACHE-II/SOFA, functional and nutritional status, lactate, vasopressor use, ventilation, chills, serial procalcitonin, previous biliary interventions and concurrent pancreatitis. ICU timing relative to drainage could not be reconstructed. Although the patient-level cross-tabulation was available and showed that all ICU admissions occurred among Tokyo grade III patients, only five grade III patients were managed without ICU. This nested distribution prevented reliable assessment of the incremental information provided by ICU admission beyond Tokyo grade and contributed to the decision not to fit a joint model. Access route, guidance modality, catheter calibre, time to drainage and procedure-related complications were not sufficiently complete for uniform reporting. Laboratory analyses were exploratory and uncorrected for multiplicity. Formal linearity-in-the-logit diagnostics and Firth penalised-logistic sensitivity models were not performed. Two patients lacked complete 90-day follow-up, although extreme-case mortality proportions and crude associations were similar. Causes of death were not reliably ascertainable, so only all-cause mortality was analysed. The cohort lacked an early-cholecystectomy comparator and external validation; findings may not generalise to centres with different referral, ICU or drainage practices.

## 6. Conclusions

In this retrospective single-centre cohort, Tokyo grade III disease and ICU admission during the index episode were strongly associated with 30- and 90-day mortality in separate age-adjusted models. Because ICU admission was nested within Tokyo grade III and its timing relative to drainage was not uniformly available, the estimates should be interpreted as overlapping severity associations rather than independent effects. Residual confounding, the limited event count and wide CIs also preclude claims of validated predictive performance. The findings may support cautious bedside risk discussion but require confirmation in larger, preferably multicentre cohorts with standardised baseline and procedural data.

## Figures and Tables

**Table 1:** Clinical characteristics according to 90-day mortality status.

Characteristic	Alive at 90 Days (n = 62)	Died within 90 Days (n = 20)	*p* Value
Age, years	73.0 (64.0–85.0)	84.0 (72.8–89.0)	0.038
Charlson comorbidity index	1.0 (0.0–1.0)	2.0 (1.0–3.0)	<0.001
Symptom duration, days	3.5 (2.0–5.0)	3.0 (2.0–5.0)	0.763
Age ≥ 80 years	19/62 (30.6)	12/20 (60.0)	0.019
Male sex	33/62 (53.2)	15/20 (75.0)	0.086
ICU admission during index episode	9/62 (14.5)	16/20 (80.0)	<0.001
Tokyo grade III disease	13/62 (21.0)	17/20 (85.0)	<0.001
Tokyo severity grade			<0.001
Grade I	9/62 (14.5)	0/20 (0.0)	
Grade II	40/62 (64.5)	3/20 (15.0)	
Grade III	13/62 (21.0)	17/20 (85.0)	
Mutually exclusive presentation category			0.442
Calculous, without perforation	44/62 (71.0)	14/20 (70.0)	
Perforated, irrespective of stone status	16/62 (25.8)	4/20 (20.0)	
Acalculous, without perforation	2/62 (3.2)	2/20 (10.0)	
Positive bile culture among cultured patients	26/53 (49.1)	6/16 (37.5)	0.417

Values are median (interquartile range) or n/N (%). Two patients who were alive with less than 90 days of follow-up were excluded from binary 90-day comparisons. Bile-culture status was available for 69/82 patients. For presentation category, documented perforation superseded stone status; non-perforated cases were classified as calculous or acalculous. Abbreviation: ICU, intensive care unit.

**Table 2:** Admission laboratory values according to 90-day mortality status.

Admission Variable	Alive at 90 Days (n = 62)	Died Within 90 Days (n = 20)	*p* Value
White cell count, ×10^9^/L	15.8 (12.8–19.7)	16.5 (10.8–24.0)	0.923
Neutrophil-to-lymphocyte ratio	11.3 (7.3–20.7)	17.3 (12.2–27.7)	0.104
C-reactive protein, mg/L	224.1 (98.7–300.5)	110.6 (68.5–185.1)	0.037
Urea, mg/dL	42.0 (26.2–67.5)	73.5 (56.0–109.5)	<0.001
Creatinine, mg/dL	1.0 (0.8–1.4)	1.3 (1.0–2.1)	0.029
Total bilirubin, mg/dL	1.2 (0.7–2.0)	2.2 (1.1–4.3)	0.033
Direct bilirubin, mg/dL	0.5 (0.4–1.0)	1.4 (0.4–3.6)	0.060

Footnotes: Values are median (interquartile range). Laboratory comparisons were exploratory; *p* values are nominal and were not adjusted for multiplicity.

**Table 3:** Thirty- and ninety-day mortality across pre-specified clinical strata.

Stratification	Group	N at 30 Days	30-Day Mortality, n/N (%)	N Evaluable at 90 Days	90-Day Mortality, n/N (%)
Tokyo grade	I	9	0/9 (0.0)	9	0/9 (0.0)
	II	44	2/44 (4.5)	43	3/43 (7.0)
	III	31	10/31 (32.3)	30	17/30 (56.7)
ICU admission during index episode	No	58	2/58 (3.4)	57	4/57 (7.0)
	Yes	26	10/26 (38.5)	25	16/25 (64.0)
Age group	<80 years	53	4/53 (7.5)	51	8/51 (15.7)
	≥80 years	31	8/31 (25.8)	31	12/31 (38.7)
Charlson index	0–1	54	5/54 (9.3)	54	7/54 (13.0)
	≥2	30	7/30 (23.3)	28	13/28 (46.4)
Admission NLR	<12.30	42	3/42 (7.1)	40	6/40 (15.0)
	≥12.30	42	9/42 (21.4)	42	14/42 (33.3)
Admission urea	<50.9 mg/dL	42	3/42 (7.1)	40	3/40 (7.5)
	≥50.9 mg/dL	42	9/42 (21.4)	42	17/42 (40.5)
Total bilirubin	<2.0 mg/dL	57	4/57 (7.0)	55	9/55 (16.4)
	≥2.0 mg/dL	27	8/27 (29.6)	27	11/27 (40.7)

Footnotes: Ninety-day denominators exclude two patients who were alive but had less than 90 days of documented follow-up. NLR and urea were dichotomised at the cohort median for descriptive presentation only. Abbreviations: ICU, intensive care unit; NLR, neutrophil-to-lymphocyte ratio.

**Table 4:** Univariable and age-adjusted logistic associations with 30-day mortality.

Factor	Scale/Comparison	Univariable OR (95% CI; *p*)	Age-Adjusted OR (95% CI; *p*)
Age	per 10-year increase	1.84 (1.07–3.17); *p* = 0.027	—
Charlson comorbidity index	per 1-point increase	2.33 (1.26–4.28); *p* = 0.007	2.50 (1.28–4.89); *p* = 0.007
Tokyo grade III disease	vs. grade I–II	12.14 (2.45–60.20); *p* = 0.002	9.44 (1.84–48.35); *p* = 0.007
ICU admission during index episode	vs. no intensive care	17.50 (3.47–88.13); *p* < 0.001	14.31 (2.77–73.99); *p* = 0.001
Admission NLR	per 5-unit increase	1.18 (1.00–1.39); *p* = 0.045	1.14 (0.97–1.35); *p* = 0.105
Admission urea	per 10 mg/dL increase	1.21 (1.05–1.40); *p* = 0.009	1.21 (1.04–1.41); *p* = 0.013
Admission creatinine	per 1 mg/dL increase	1.92 (1.02–3.64); *p* = 0.044	1.85 (0.93–3.64); *p* = 0.077
Total bilirubin	per 1 mg/dL increase	1.07 (0.96–1.19); *p* = 0.213	1.08 (0.97–1.20); *p* = 0.177

Footnote: Age-adjusted models included age as a continuous variable and one factor at a time. Abbreviations: OR, odds ratio; CI, confidence interval; ICU, intensive care unit; NLR, neutrophil-to-lymphocyte ratio.

**Table 5:** Univariable and age-adjusted logistic associations with 90-day mortality.

Factor	Scale/Comparison	Univariable OR (95% CI; *p*)	Age-Adjusted OR (95% CI; *p*)
Age	per 10-year increase	1.46 (0.99–2.14); *p* = 0.055	—
Charlson comorbidity index	per 1-point increase	3.00 (1.68–5.37); *p* < 0.001	2.95 (1.64–5.33); *p* < 0.001
Tokyo grade III disease	vs. grade I–II	21.36 (5.42–84.16); *p* < 0.001	19.07 (4.74–76.66); *p* < 0.001
ICU admission during index episode	vs. no intensive care	23.56 (6.40–86.76); *p* < 0.001	21.10 (5.65–78.85); *p* < 0.001
Admission NLR	per 5-unit increase	1.10 (0.95–1.27); *p* = 0.205	1.07 (0.92–1.24); *p* = 0.374
Admission urea	per 10 mg/dL increase	1.19 (1.04–1.35); *p* = 0.010	1.17 (1.03–1.34); *p* = 0.019
Admission creatinine	per 1 mg/dL increase	1.96 (1.09–3.54); *p* = 0.025	1.81 (1.00–3.28); *p* = 0.049
Total bilirubin	per 1 mg/dL increase	1.04 (0.94–1.15); *p* = 0.442	1.04 (0.94–1.15); *p* = 0.490

Footnote: Binary 90-day logistic analyses used 82 evaluable patients. Abbreviations: OR, odds ratio; CI, confidence interval; ICU, intensive care unit; NLR, neu-trophil-to-lymphocyte ratio.

**Table 6:** Extreme-case sensitivity analysis for incomplete 90-day status.

Scenario	90-Day Mortality, n/N (%) [95% CI]	Tokyo Grade III vs. I-II, Crude OR (95% CI)	ICU vs. no ICU, Crude OR (95% CI)
Available-case analysis	20/82 (24.4) [16.4–34.7]	21.36 (5.42–84.16)	23.56 (6.40–86.76)
Both incomplete cases assumed alive	20/84 (23.8) [16.0–33.9]	20.24 (5.18–79.09)	21.60 (5.97–78.22)
Both incomplete cases assumed dead	22/84 (26.2) [18.0–36.5]	16.96 (4.89–58.85)	20.02 (5.90–67.96)

Footnotes: Odds ratios are unadjusted. Of the two patients with incomplete 90-day status, one had Tokyo grade III disease and ICU admission, whereas the other had Tokyo grade II disease and no ICU admission. Abbreviations: OR, odds ratio; CI, confidence interval; ICU, intensive care unit.

## Data Availability

The individual-level datasets generated and analysed in the current study are not publicly available due to patient confidentiality and institutional data protection requirements. An anonymised aggregate dataset and analysis code may be made available from the corresponding author on reasonable request, subject to institutional approval.

## Supplementary Materials

The following supporting information can be downloaded at: https://www.mdpi.com/article/10.3390/jcm15166270/s1, File S1: STROBE checklist for the retrospective cohort study. Table S1: Patient-level cross-tabulation of Tokyo severity grade and ICU admission during the index episode. Figure S1: Thirty- and ninety-day mortality according to Tokyo severity grade.

## References

[1] Sadaka A.H., Tseng J.F., Itani K.M.F. (2025). Indications for and Optimal Management of Percutaneous Cholecystostomy Drainage: A Systematic Review. JAMA Surg..

[2] Huang R., Patel D.C., Kallini J.R., Wachsman A.M., Van Allan R.J., Margulies D.R., Phillips E.H., Barmparas G. (2022). Percutaneous Cholecystostomy Tube for Acute Cholecystitis: Quantifying Outcomes and Prognosis. J. Surg. Res..

[3] Latif J., Kushairi A., Thurley P., Bhatti I., Awan A. (2022). Laparoscopic Cholecystectomy versus Percutaneous Cholecystostomy: Suitability of APACHE-II Score, ASA Grade, and Tokyo Guidelines 18 Grade as Predictors of Outcome in Patients with Acute Cholecystitis. Surg. Laparosc. Endosc. Percutan. Tech..

[4] Rubio-García J.J., Vico D.V., Tudela C.V., López J.I., Padilla D.C., López C.A., Morote S.C., Ramia-Ángel J.M. (2023). Impact of Percutaneous Cholecystostomy in the Management of Acute Cholecystitis: A Retrospective Cohort Study at a Tertiary Center. Updat. Surg..

[5] Doğrul A.B., Oruç M., Ciftci T., Hayran K.M., Abbasoğlu O. (2022). Factors Affecting Interval Cholecystectomy and Mortality in Percutaneous Cholecystostomy Patients. Turk. J. Trauma Emerg. Surg..

[6] Teke E., Agca B., Güneş Y., Teke G.N., Yaz A.S., Aydin M.T., Yıldırım G. (2025). Percutaneous Cholecystostomy in Elderly Patients with Acute Cholecystitis: Factors Influencing Mortality, Morbidity, and Length of Hospital Stay. Turk. J. Trauma Emerg. Surg..

[7] Yodying H., Somtasana K., Toemakharathaworn K. (2025). Neutrophil Percentage-to-Albumin Ratio as a Predictor of Conservative Treatment Failure in Acute Cholecystitis: A Retrospective Cohort Study. BMC Surg..

[8] Fanciulli G., Favara G., Maugeri A., Barchitta M., Agodi A., Basile G. (2025). Comparing Percutaneous Treatment and Cholecystectomy Outcomes in Acute Cholecystitis Patients: A Systematic Review and Meta-analysis. World J. Emerg. Surg..

[9] Acker R.C., Ginzberg S.P., Sharpe J., Keele L., Hwang J., Bakillah E., Goldberg D., Kaufman E., Kelz R.R. (2025). Operative vs. Nonoperative Treatment of Acute Cholecystitis in Older Adults with Multimorbidity. JAMA Surg..

[10] Gross A.R., Littau M., Wehrle C.J., Slaibi A., Leo R., Said S.A.-D., Simon R., Joyce D., Asfaw S.H., Walsh R.M. (2025). Reducing Percutaneous Cholecystostomy for Acute Calculous Cholecystitis: A Multisite Quality Improvement Initiative. Surgery.

[11] Ghali M.S., Ali S.M., Gibreal K.J.S., Singh R., Shehata M.S., Al-Zoubi R.M., Zarour A. (2025). Indications and Clinical Outcomes of Percutaneous Cholecystostomies in Acute Cholecystitis: A Study from Qatar. BMC Surg..

[12] Slomowitz E., Cooper L., Tsivion-Visbord H., Shochat T., Kashtan H., Schrier I. (2025). Outcomes of Percutaneous Cholecystostomy in Older Adults with Acute Cholecystitis. Isr. Med. Assoc. J..

[13] Curry J., Chervu N., Cho N.Y., Hadaya J., Vadlakonda A., Kim S., Keeley J., Benharash P. (2024). Percutaneous Cholecystostomy Tube Placement as a Bridge to Cholecystectomy for Grade III Acute Cholecystitis: A National Analysis. Surg. Open Sci..

[14] Podder S., Lung K., Ibrahim G., Koeneman S., Marks J., Cohen M., Kohli A. (2025). Barriers to Interval Cholecystectomy following Percutaneous Cholecystostomy in Patients with Acute Calculous Cholecystitis. Surg. Endosc..

[15] Lin M.-H., Ni C.-F., Chiang H.-J., Chen Y.-T., Tsai C.-C., Chen Y.-C., Huang S.-W., Chen Y.-T., Wu J.-C., Cheng S.-J. (2025). Optimal Timing of Percutaneous Cholecystostomy across Different Grades of Acute Cholecystitis: A Retrospective Cohort Study. J. Vasc. Interv. Radiol..

[16] Pesce A.M., Ramírez-Giraldo C., Arkoudis N.-A., Ramsay G., Popivanov G., Gurusamy K., Bejarano N., Bellini M.I., Allegritti M., Tesei J. (2025). Management of High-Surgical-Risk Patients with Acute Cholecystitis following Percutaneous Cholecystostomy: Results of an International Delphi Consensus Study. Int. J. Surg..

