# Supplementary material for: Factors Associated with 30- and 90-Day Mortality After Percutaneous Cholecystostomy for Acute Cholecystitis: A Single-Centre Retrospective Cohort Study
Source: J Clin Med. 2026 Aug 13;15(16):6270. doi: 10.3390/jcm15166270 (PMC13513208; doi:10.3390/jcm15166270)
Supplement: Supplementary file 1 [file jcm-15-06270-s001.zip › jcm-4453916-File S1.pdf]

STROBE Statement Checklist for Cohort Studies

Manuscript: Prognostic Factors for 30- and 90-Day Mortality After Percutaneous Cholecystostomy for Acute Cholecystitis: A Single-Centre Retrospective Cohort Study

Item	Recommendation	Location in manuscript
1(a)	Indicate the study design with a commonly used term in the title or abstract.	Title; Abstract; Materials and Methods, Study Design and Setting
1(b)	Provide an informative and balanced summary of what was done and what was found.	Abstract
2	Explain the scientific background and rationale for the investigation.	Introduction
3	State specific objectives, including any prespecified hypotheses.	End of Introduction
4	Present key elements of study design early in the paper.	Materials and Methods, Study Design and Setting
5	Describe the setting, locations and relevant dates, including periods of recruitment, exposure, follow-up and data collection.	Materials and Methods, Study Design and Setting
6(a)	Give eligibility criteria and methods of selection of participants.	Materials and Methods, Patient Selection
6(b)	For matched studies, give matching criteria and numbers of exposed/unexposed.	Not applicable
7	Clearly define all outcomes, exposures, predictors, confounders and effect modifiers.	Materials and Methods, Outcomes and Candidate Prognostic Variables
8	For each variable of interest, give sources of data and details of assessment.	Materials and Methods, Data Collection
9	Describe efforts to address potential sources of bias.	Materials and Methods; Discussion, Strengths and Limitations
10	Explain how study size was arrived at.	Materials and Methods, Statistical Analysis
11	Explain how quantitative variables were handled in the analyses.	Materials and Methods, Statistical Analysis; Tables 3–5
12(a)	Describe all statistical methods, including those used to control for confounding.	Materials and Methods, Statistical Analysis
12(b)	Describe methods used to examine subgroups and interactions.	Materials and Methods, Statistical Analysis
12(c)	Explain how missing data were addressed.	Materials and Methods, Statistical Analysis; Results
12(d)	Explain how loss to follow-up was addressed.	Materials and Methods, Outcomes; Results
12(e)	Describe sensitivity analyses.	Materials and Methods, Outcomes
13(a)	Report numbers of individuals at each stage of the study.	Results; Table 1
13(b)	Give reasons for non-participation at each stage.	Patient selection; not applicable after cohort identification
13(c)	Consider use of a flow diagram.	Not used; cohort was consecutive with no exclusions after eligibility screening
14(a)	Give characteristics of study participants and information on exposures and confounders.	Results; Table 1
14(b)	Indicate number of participants with missing data for each variable of interest.	Results; table footnotes and denominators
14(c)	Summarise follow-up time.	Results
15	Report numbers of outcome events or summary measures over time.	Results; Tables 1 and 3; Figure 3
16(a)	Give unadjusted estimates and, if applicable, confounder-adjusted estimates with precision.	Results; Tables 4 and 5; Figure 2
16(b)	Report category boundaries when continuous variables were categorised.	Table 3 footnote
16(c)	Consider translating estimates of relative risk into	Results; Table 3

Item	Recommendation	Location in manuscript
	absolute risk.	
17	Report other analyses such as subgroup and sensitivity analyses.	Results; Tables 3–5
18	Summarise key results with reference to study objectives.	Discussion
19	Discuss limitations, including sources of potential bias or imprecision.	Strengths and Limitations
20	Give a cautious overall interpretation of results.	Discussion; Conclusions
21	Discuss generalisability of the results.	Strengths and Limitations
22	Give the source of funding and role of funders.	Funding
