# Supplementary material for: Factors Associated with 30- and 90-Day Mortality After Percutaneous Cholecystostomy for Acute Cholecystitis: A Single-Centre Retrospective Cohort Study
Source: J Clin Med. 2026 Aug 13;15(16):6270. doi: 10.3390/jcm15166270 (PMC13513208; doi:10.3390/jcm15166270)
Supplement: Supplementary file 1 [file jcm-15-06270-s001.zip › jcm-4453916-supplementary.pdf]

Supplementary Table S1

Factors Associated with 30- and 90-Day Mortality After Percutaneous Cholecystostomy for Acute Cholecystitis: A Single-Centre Retrospective Cohort Study

Table S1. Patient-level cross-tabulation of Tokyo severity grade and ICU admission during the index episode.

Tokyo severity grade	No ICU admission	ICU admission	Total
Grade I	9	0	9
Grade II	44	0	44
Grade III	5	26	31
**Total**	**58**	**26**	**84**

ICU admission was defined as any admission to intensive care during the index acute-care episode. All 26 ICU admissions occurred among Tokyo grade III patients; 26 of 31 grade III patients (83.9%) entered ICU. The timing of ICU admission relative to percutaneous cholecystostomy was not reproducibly available.

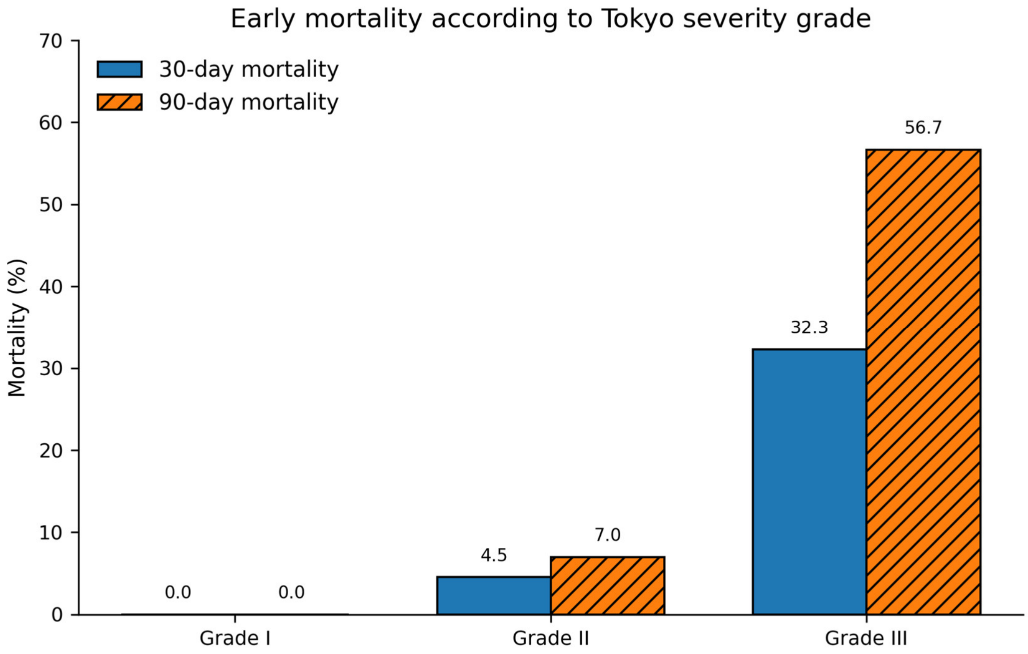

Figure S1. Thirty- and ninety-day mortality according to Tokyo severity grade.
